# Synergistic Alleviation of Inflammatory Cytokine Storms in Sepsis Rats by Low‐Intensity Pulsed Ultrasound and Imipenem

**DOI:** 10.1155/mi/7323319

**Published:** 2026-01-16

**Authors:** Wentao Tang, Juan Deng, Xinyi Zhang, Guolin Song, Juan Qin, Chenghai Li, Xinfang Xiao, Liu Wu, Yilin Tang, Yiqing Zhou, Junfen Li, Sicheng He, Yan Wang

**Affiliations:** ^1^ State Key Laboratory of Ultrasound in Medicine and Engineering, College of Biomedical Engineering, Chongqing Medical University, Chongqing, 400016, China, cqmu.edu.cn; ^2^ Department of Emergency, The Second Affiliated Hospital of Guizhou University of Traditional Chinese Medicine, Guizhou, 550003, China, gzu.edu.cn; ^3^ Department of Obstetrics and Gynecology, Guiyang Maternal and Child Health Care Hospital, Guizhou Medical University, Guizhou, 550003, China, gmc.edu.cn

**Keywords:** IL-1R/NF-*κ*B signaling pathway, IMI, inflammation, low-intensity ultrasound, sepsis

## Abstract

**Aims:**

This study aimed to investigate the effects of low intensity pulsed ultrasound (LIPUS) combined with imipenem (IMI) on inflammatory responses and organ protection in septic rats.

**Results:**

The study involved 230 Sprague–Dawley (SD) rats, with 80 used for survival analysis and 150 for sampling over 72 h. Histological examination (hematoxylin and eosin [H&E] staining) and transmission electron microscopy (TEM) revealed that LIPUS combined with IMI significantly alleviated spleen tissue damage and reduced mitochondrial edema. Key inflammatory cytokines, such as interleukin‐1 beta (IL‐1*β*), tumor necrosis factor‐alpha (TNF‐*α*), and IL‐6 were significantly decreased, while IL‐10 levels increased in the LIPUS + IMI group (*p* < 0.05). The combined treatment also reduced the expression of cytokines, such as IL‐1 receptor (IL‐1R), nuclear factor kappa B p65 (NF‐*κ*B p65), transforming growth factor‐beta (TGF‐*β*), and high mobility group protein box 1 (HMGB1), indicating a reduction in inflammation (*p* < 0.05).

**Conclusion and Innovation:**

This study presents a novel approach by integrating LIPUS with IMI, providing a noninvasive and effective strategy to mitigate cytokine storms, optimize antibiotic use, and reduce organ damage in sepsis. The protective effects observed are primarily attributed to the inhibition of the IL‐1R/NF‐*κ*B signaling pathway, which significantly improves survival outcomes in septic rats. This combined therapy has potential for enhancing sepsis treatment protocols.

## 1. Introduction

In the latest “Sepsis‐3” consensus definition, sepsis is defined as a life‐threatening dysfunction of organs caused by an unregulated host response to infection [1]. A hallmark of sepsis pathophysiology is its biphasic immune response, characterized by both an excessive pro‐inflammatory “cytokine storm” and subsequent immunosuppression, which disrupts the physiological homeostasis of vital organs, including the kidneys, liver, lungs, and heart, ultimately leading to multiple organ dysfunction syndrome (MODS) [2]. MODS is a major cause of death among ICU patients.

The pathological process of sepsis involves a complex inflammatory response, with the cytokine storm being a critical mechanism. The cytokine storm refers to the excessive activation of the immune system following infection, resulting in the massive release of various inflammatory cytokines, such as tumor necrosis factor‐alpha (TNF‐*α*), interleukin (IL)‐1*β*, and IL‐6 [3, 4]. However, the urgent nature of sepsis often hinders optimal selection and dosage determination for antibiotics [5], which can elevate the risk of adverse events, organ dysfunction, or even mortality during treatment [6].

Antibiotic therapy remains the cornerstone of sepsis management, and timely administration can significantly reduce morbidity and mortality [7]. Imipenem (IMI), a broad‐spectrum *β*‐lactam, is widely used in clinical practice and has been shown to reduce inflammatory cytokines such as IL‐6 and TNF‐*α* in septic models [8, 9]. In practice, IMI is administered every 6–8 h [10, 11]. However, its prolonged or excessive use can disrupt immune homeostasis and cause organ toxicity, highlighting the need for strategies to optimize antibiotic efficacy while minimizing adverse effects.

Low intensity pulsed ultrasound (LIPUS), a noninvasive physical therapy that offers several advantages, including noninvasiveness, portability, and strong targeting capabilities [12]. LIPUS primarily relies on nonthermal mechanical effects to influence cellular signaling pathways, including NF‐*κ*B and MAPK, which can suppress inflammatory responses [13–15]. Importantly, targeting the spleen with LIPUS may modulate systemic immune activity, as the spleen plays a central role in balancing inflammation and immunosuppression during sepsis. Our previous research further demonstrated that LIPUS irradiation of the spleen in septic rats significantly reduced IL‐1*β* and TNF‐*α* levels and improved survival rates [16]. Its low cost and minimal risk make LIPUS a promising adjunctive therapy in inflammatory diseases [15, 17–20]. Based on these findings, we hypothesized that the combination of LIPUS and IMI could synergistically regulate the dysregulated immune response in sepsis by suppressing excessive proinflammatory cytokine release while alleviating subsequent immunosuppression. This study aimed to evaluate the effects of LIPUS combined with IMI on survival, cytokine storm regulation, and organ protection in septic rats, and to explore potential underlying mechanisms. Given sepsis’ global burden, particularly in resource‐limited regions, this research may provide a foundation for developing noninvasive, cost‐effective adjunctive therapies.

## 2. Results

### 2.1. Timing of Administration of IMI

Our study revealed significant differences in PCT and CRP levels between the experimental groups. As shown in Figure 1A, PCT levels in the CLP group exhibited a continuous upward trend, peaking at 24 h. In contrast, the LIPUS group demonstrated significantly lower PCT levels at 4, 8, 16, 24, 48, and 72 h compared to the CLP group (*p* < 0.05). Similarly, Figure 1B indicates that CRP levels in the CLP group increased until reaching a maximum at 48 h. The LIPUS group, however, displayed a significant decrease in CRP levels at all examined time points (4–72 h) compared to the CLP group (*p* < 0.05). These findings suggest that LIPUS can effectively modulate inflammation within the 4–72 h timeframe. Based on these observations, we developed an optimized clinical regimen for IMI administration: two doses, given every 24 h, with the first dose administered 1 h post‐CLP surgery.

Figure 1Effects of LIPUS combined with IMI on septic rats. (A, B) Effects of LIPUS on the expression levels of PCT and CRP.  ^∗^ *p* < 0.05. (C, D) Effects of LIPUS on 72 h survival rate and survival time in CLP, LIPUS, IMI, LIPUS + IMI, and Sham group.  ^&^ *p* < 0.05 vs. IMI group;  ^∗^ *p* < 0.05 vs. Sham group;  ^#^ *p* < 0.05 vs. CLP group. (E) Representative HE staining of spleen from CLP, LIPUS, IMI, LIPUS + IMI, and Sham groups (×100). (F) The ultrastructure changes of spleen tissue in the CLP, LIPUS, IMI, LIPUS + IMI, and Sham groups was observed by TEM (the green arrows represent mitochondrial edema, the blue arrows represent endoplasmic reticulum dilation, and the red arrows represent remission of mitochondrial edema).

**Figure:**
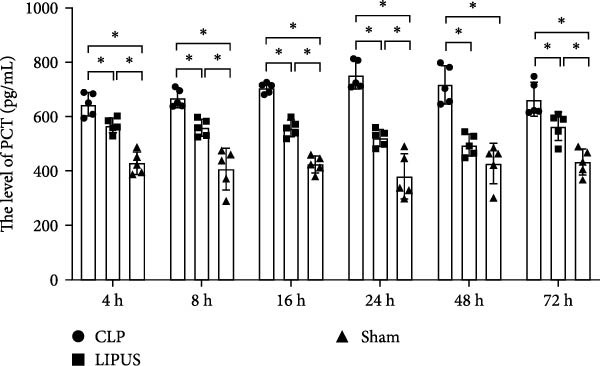
(A)

**Figure:**
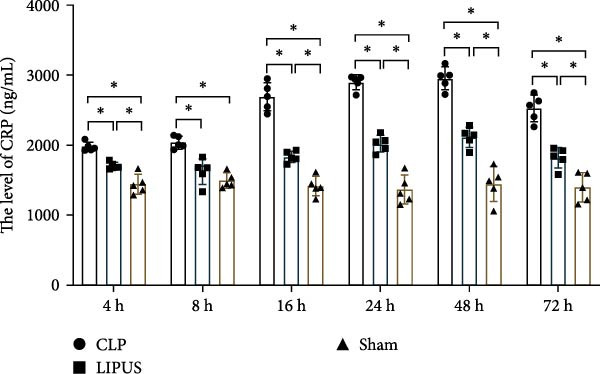
(B)

**Figure:**
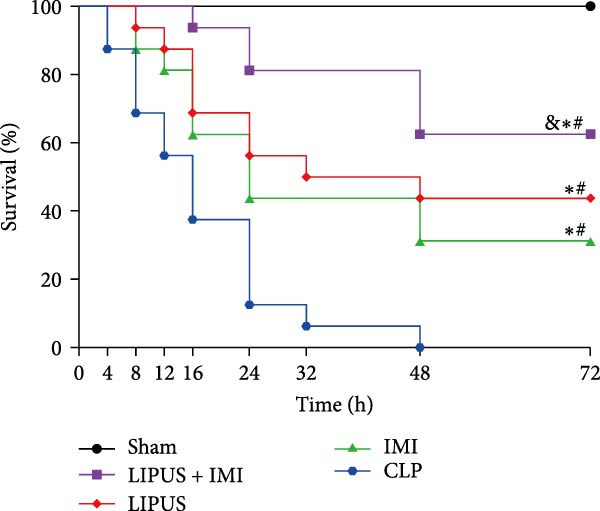
(C)

**Figure:**
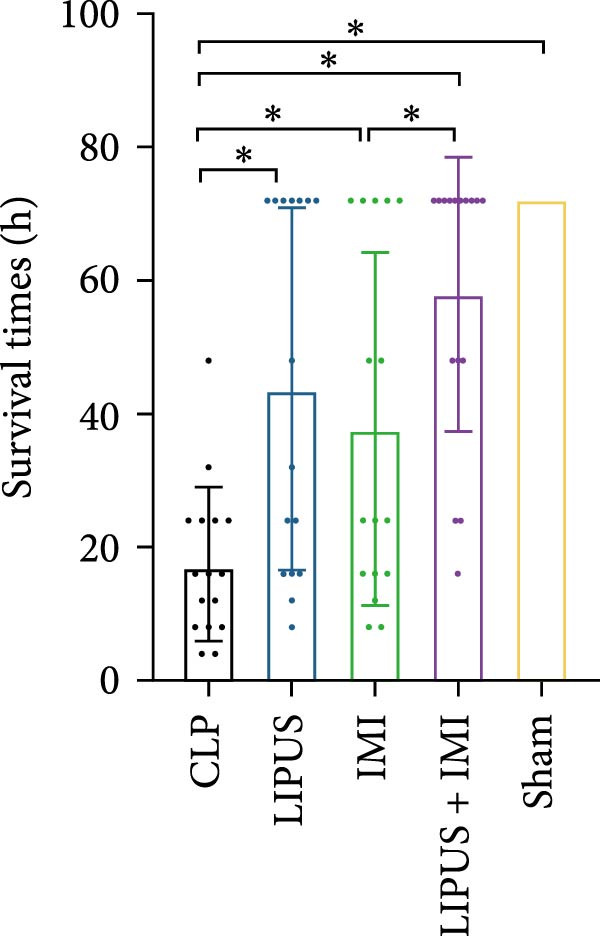
(D)

**Figure:**
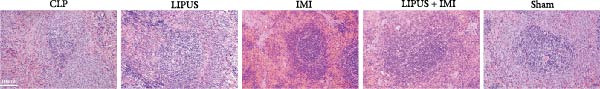
(E)

**Figure:**
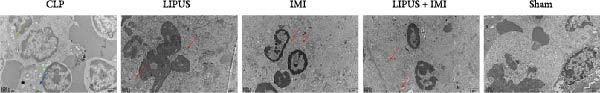
(F)

### 2.2. The Impact of LIPUS Combined With IMI on 72‐h Survival Rate

The successful establishment of the sepsis model was indicated by reduced motor activity, lethargy, chills, and piloerection [21]. In the IMI group, 5 out of 16 rats survived within 72 h, resulting in a survival rate of 31.25% and a survival time of 37.75 ± 26.45 h. The LIPUS group had seven survivors, with a survival rate of 43.75% and a survival time of 43.75 ± 27.23 h. The combination therapy group (LIPUS + IMI) had the highest survival rate, with 10 out of 16 rats surviving (62.50%) and a survival time of 58.00 ± 20.55 h. All rats in the CLP group died within 72 h, with a survival time of 17.50 ± 11.58 h, while all rats in the Sham group survived the entire 72‐h period. Both the IMI and LIPUS groups significantly improved survival rates compared to the CLP group. Notably, the LIPUS + IMI group showed a significant improvement in both survival rate (*p* < 0.05; Figure 1C) and survival time (*p* < 0.05; Figure 1D) compared to the IMI group alone.

### 2.3. The Impact of LIPUS Combined With IMI on Spleen Histopathology

The boundary between the white and red pulp of the spleen tissue in the CLP group was indistinct, as depicted in Figure 1E, with evident infiltration of inflammatory cells. In contrast, the LIPUS group, IMI group, and LIPUS + IMI group exhibited a more distinct demarcation between white pulp and red pulp in the spleen, accompanied by reduced inflammatory cell infiltration. The Sham group displayed normal structure of both white pulp and red pulp.

### 2.4. The Impact of LIPUS Combined With IMI on the Spleen’s Ultrastructure

The CLP group exhibited mitochondrial edema (indicated by the green arrow), endoplasmic reticulum dilation (indicated by the blue arrow), and perinuclear space dilation, as depicted in Figure 1F. However, in the LIPUS, IMI, and LIPUS + IMI groups, relief of mitochondrial edema was observed (indicated by the red arrow). Notably, the spleen ultrastructure remained normal in the Sham group.

### 2.5. Impact of LIPUS Combined With IMI on Cytokine Levels in Serum and Spleen

#### 2.5.1. Serum Cytokine Levels

As shown in Figure 2A, the serum levels of IL‐1*β* at 8, 24, 48, and 72 h were significantly lower in the LIPUS, IMI, and LIPUS + IMI groups compared to the CLP group (*p* < 0.05). Figure 2B illustrates that serum TNF‐*α* levels at 4, 8, 16, 24, 48, and 72 h post‐treatment were significantly reduced in the LIPUS, IMI, and LIPUS + IMI groups compared to the CLP group (*p* < 0.05). Additionally, at all time points except 48 h, both the IMI and LIPUS + IMI groups exhibited significantly lower TNF‐*α* levels than the LIPUS group alone (*p* < 0.05). Figure 2C indicates that serum IL‐6 levels at all measured time points (4–72 h) were significantly lower in the LIPUS and IMI groups compared to the CLP group (*p* < 0.05). Furthermore, from 16 to 72 h, the LIPUS + IMI group showed a significantly greater decrease in IL‐6 levels compared to the IMI group alone (*p* < 0.05).

Figure 2Effect of LIPUS combined with IMI on cytokine levels in serum and spleen. (A–F) Effects of LIPUS combined with IMI on the expression levels of IL‐1*β*, TNF‐*α*, IL‐6, HMGB‐1, TGF‐*β*, and IL‐10 in serum. (G–L) Effects of LIPUS combined with IMI on the expression levels of IL‐1*β*, TNF‐*α*, IL‐6, HMGB‐1, TGF‐*β*, and IL‐10 in spleen.  ^∗^ *p* < 0.05.

**Figure:**
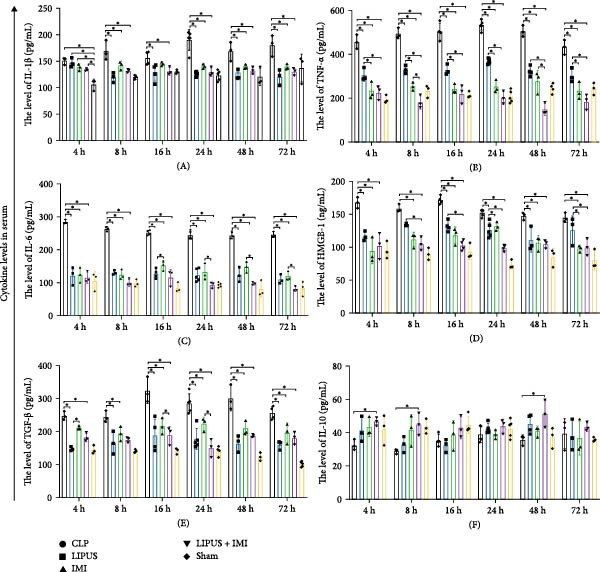

**Figure:**
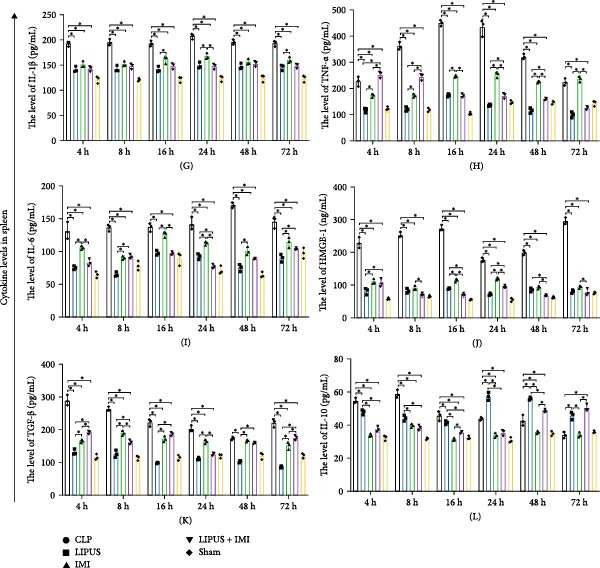

In Figure 2D, serum high mobility group protein box 1 (HMGB1) levels in the LIPUS, IMI, and LIPUS + IMI groups were significantly lower than those in the CLP group at 4, 16, and 48 h (*p* < 0.05). Additionally, at 8, 16, 24, and 72 h, HMGB1 levels in the LIPUS + IMI group were significantly lower compared to the LIPUS group alone (*p* < 0.05). Figure 2E shows that serum transforming growth factor‐beta (TGF‐*β*) levels were significantly lower in the LIPUS and IMI groups compared to the CLP group at 16, 24, 48, and 72 h (*p* < 0.05). At 24 h, the LIPUS + IMI group had significantly lower TGF‐*β* levels than the IMI group alone (*p* < 0.05). Figure 2F indicates that serum IL‐10 levels were significantly elevated at 4, 8, and 48 h in the LIPUS + IMI group compared to the CLP group (*p* < 0.05).

#### 2.5.2. Spleen Cytokine Levels

Figure 2G shows that IL‐1*β* levels in the spleen were significantly lower in the LIPUS, IMI, and LIPUS + IMI groups compared to the CLP group at all time points (4–72 h) (*p* < 0.05). Additionally, at 24 h, IL‐1*β* levels were significantly lower in both the LIPUS and LIPUS + IMI groups compared to the IMI group (*p* < 0.05). Figure 2H illustrates that TNF‐*α* levels in the spleen were significantly lower in the LIPUS, IMI, and LIPUS + IMI groups compared to the CLP group at 4, 8, 16, 24, and 48 h (*p* < 0.05). At 16, 24, 48, and 72 h, TNF‐*α* levels were significantly lower in the LIPUS and LIPUS + IMI groups compared to the IMI group (*p* < 0.05).

Figure 2I demonstrates that IL‐6 levels in the spleen were significantly lower in the LIPUS, IMI, and LIPUS + IMI groups compared to the CLP group at 4, 8, 24, 48, and 72 h (*p* < 0.05). At 4, 16, and 24 h, IL‐6 levels were significantly lower in the LIPUS + IMI group compared to the IMI group (*p* < 0.05). Figure 2J shows that HMGB1 levels in the spleen were significantly lower in the LIPUS, IMI, and LIPUS + IMI groups compared to the CLP group at all time points (4–72 h) (*p* < 0.05). The LIPUS + IMI group had significantly lower HMGB1 levels than the IMI group at 8, 16, 24, 48, and 72 h (*p* < 0.05).

Figure 2K illustrates that TGF‐*β* levels in the spleen were significantly lower in the LIPUS, IMI, and LIPUS + IMI groups compared to the CLP group at 4, 8, 16, 24, and 72 h (*p* < 0.05). At 8 and 24 h, TGF‐*β* levels in the LIPUS + IMI group were significantly lower compared to the IMI group (*p* < 0.05). Figure 2L shows that IL‐10 levels in the spleen were significantly higher in the LIPUS, IMI, and LIPUS + IMI groups compared to the CLP group at 4, 8, 16, 24, 48, and 72 h (*p* < 0.05). Additionally, at 16, 48, and 72 h, IL‐10 levels in the LIPUS + IMI group were significantly higher than those in the IMI group (*p* < 0.05).

### 2.6. Immunohistochemical Analysis of the Levels of Cytokines

The expression levels of TNF‐*α* in the spleen of the LIPUS group, IMI group, and LIPUS + IMI group were significantly lower than those of the CLP group at 4, 16, 24, 48, and 72 h (*p* < 0.05), as depicted in Figure 3A. At 8, 16, 48, and 72 h (*p* < 0.05), the expression of TNF‐*α* in the spleen was significantly lower in the LIPUS + IMI group compared to the IMI group. Additionally, at 24 h (*p* < 0.05), the expression of TNF‐*α* in the spleen was significantly lower in the LIPUS + IMI group compared to the LIPUS group. The expression level of nuclear factor kappa B p65 (NF‐*κ*B p65) in the spleen of the LIPUS + IMI group was significantly lower than that in the LIPUS group and IMI group at the aforementioned time points (*p* < 0.05). As shown in Figure 3C, at 4, 8, 16, 24, 48, and 72 h, the expression levels of IL‐1*β* in the spleen were significantly lower in the LIPUS group, IMI group, and LIPUS + IMI group compared to those in the CLP group (*p* < 0.05). At these time points as well as at 24 h post‐treatment, the expression levels of IL‐1*β* were significantly lower in the spleen of the LIPUS + IMI group compared to both LIPUS and IMI groups (*p* < 0.05), with a significant difference observed between LIPUS + IMI and IMI groups specifically at 24 h post‐treatment (*p* < 0.05). Furthermore, as depicted in Figure 3D, at 4, 16, 24, 48, and 72 h after treatment initiation, the expression levels of IL‐6 in the spleen were markedly reduced in the LIPUS group, IMI group, and LIPUS + IMI group compared to the CLP group (*p* < 0.05).

Figure 3Immunohistochemical analysis of the levels of cytokines. Quantitative analysis of TNF‐*α* (A), NF‐*κ*B p65 (B), IL‐1*β* (C), IL‐6 (D), and IL‐10 (E) in the spleen of the CLP, LIPUS, IMI, LIPUS + IMI, and Sham group.  ^∗^ *p* < 0.05.

**Figure:**
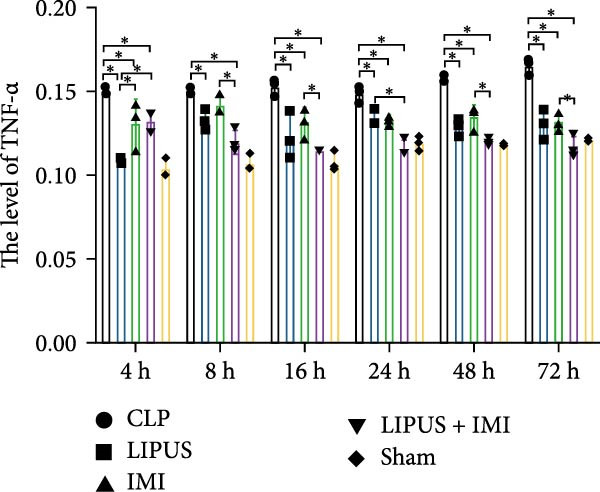
(A)

**Figure:**
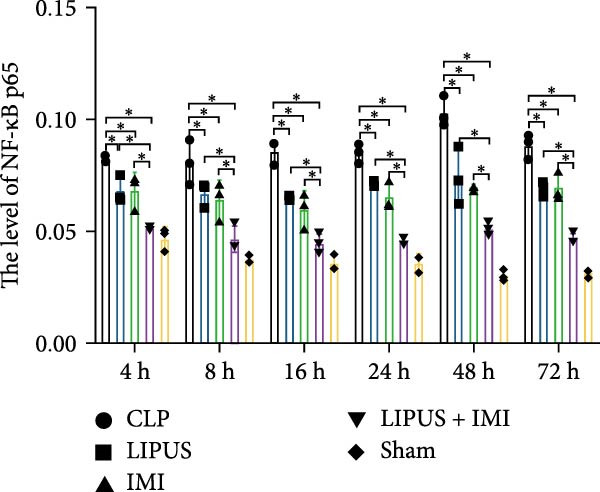
(B)

**Figure:**
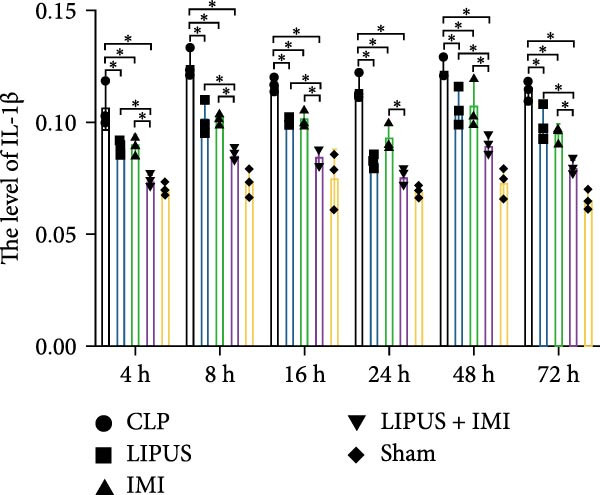
(C)

**Figure:**
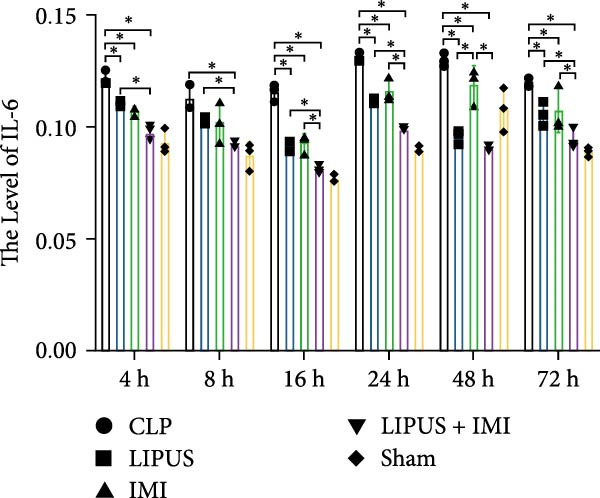
(D)

**Figure:**
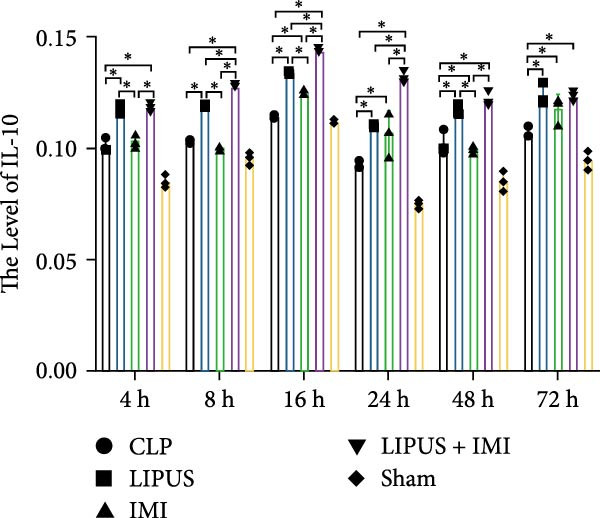
(E)

At 4 and 8 h, the expression levels of IL‐6 in the spleen of the LIPUS + IMI group was significantly lower than that of LIPUS group (*p* < 0.05). At 16, 24, and 72 h, the expression level of IL‐6 in the spleen of LIPUS + IMI group was significantly lower than those of LIPUS group and IMI group (*p* < 0.05). At 48 h, the expression levels of IL‐6 in spleen of LIPUS group and LIPUS + IMI group were significantly lower than those of IMI group (*p* < 0.05). As shown in Figure 3E, at 16, 24, 48 and 72 h, the expression levels of IL‐10 in the spleen of the LIPUS group, IMI group, and LIPUS + IMI group were significantly higher than those of the CLP group (*p* < 0.05). At 4, 8, 16, and 48 h, the expression levels of IL‐10 in the spleen of LIPUS group and LIPUS + IMI group were significantly higher than those of IMI group (*p* < 0.05). At 24 h, the expression levels of IL‐10 in the spleen of LIPUS + IMI group were significantly higher than those of LIPUS group and IMI group (*p* < 0.05).

The potential role of the IL‐1R/NF‐*κ*B signaling pathway in LIPUS combined with IMI in inhibiting inflammation. As shown in Figure 4, the protein expression levels of TNF‐*α*, IL‐1*β*, IL‐1R, NF‐*κ*B p65, IL‐6, TGF‐*β*, and HMGB1 in the LIPUS group, IMI group, and LIPUS + IMI group were significantly lower than those in the CLP group (*p* < 0.05). The protein expression levels of TNF‐*α* and IL‐6 in LIPUS + IMI group were significantly lower than those in LIPUS group and IMI group (*p* < 0.05). The protein expression level of IL‐1R in LIPUS + IMI group was significantly lower than that in LIPUS group (*p* < 0.05). The protein expression levels of HMGB1 and TGF‐*β* in the spleen of IMI group and LIPUS + IMI group were significantly lower than those of LIPUS group (*p* < 0.05). The expression of IL‐10 in LIPUS group, IMI group, and LIPUS + IMI group was significantly higher than that in CLP group (*p* < 0.05).

Figure 4Verification of the role of the IL‐1R/ NF‐*κ*B signaling pathway. Western blots (A) and quantitative analysis of TNF‐*α*, IL‐1*β*, IL‐1R, NF‐*κ*B p65, IL‐6, TGF‐*β*, HMGB‐1, and IL‐10 in CLP, LIPUS, IMI, LIPUS + IMI, and Sham group (B).  ^∗^ *p* < 0.05.

**Figure:**
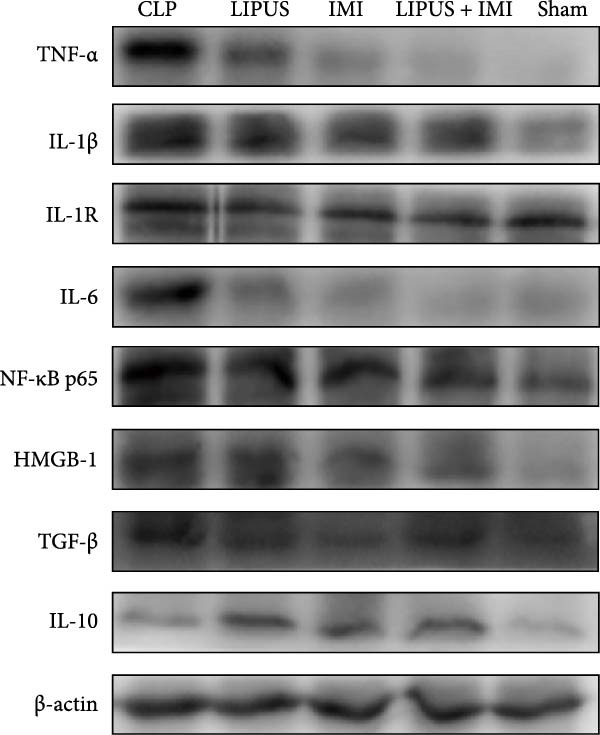
(A)

**Figure:**
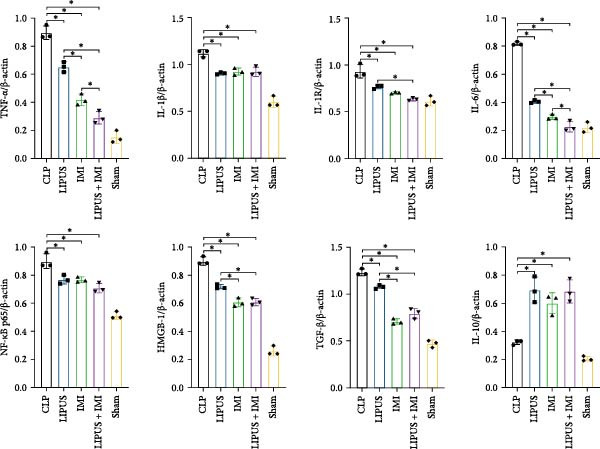
(B)

### 2.7. Relative mRNA Expression Levels of Inflammatory Cytokines

As shown in Figure 5, the mRNA expression levels of TNF‐*α*, IL‐1*β*, IL‐6, and TGF‐*β* in the LIPUS group, IMI group, and LIPUS + IMI group were significantly lower than those in the CLP group (*p* < 0.05), and the mRNA expression levels of TNF‐*α*, IL‐1*β*, and TGF‐*β* in the LIPUS + IMI group were significantly lower than those in LIPUS group and IMI group (*p* < 0.05). The expression level of IL‐6 mRNA in LIPUS + IMI group was significantly lower than that in LIPUS group (*p* < 0.05). The expression level of IL‐10 mRNA in the LIPUS group and LIPUS + IMI group was significantly higher than that in the CLP group (*p* < 0.05), and the expression level of IL‐10 mRNA in the LIPUS + IMI group was significantly higher than those in the LIPUS group and IMI group (*p* < 0.05).

Figure 5Relative mRNA expression levels of inflammatory cytokines in the spleen. (A) TNF‐*α*, (B) IL‐1*β*, (C) IL‐6, (D) TGF‐*β*, and (E) IL‐10 mRNA expression levels in the spleen of CLP, LIPUS, IMI, LIPUS + IMI, and Sham groups.  ^∗^ *p* < 0.05.

**Figure:**
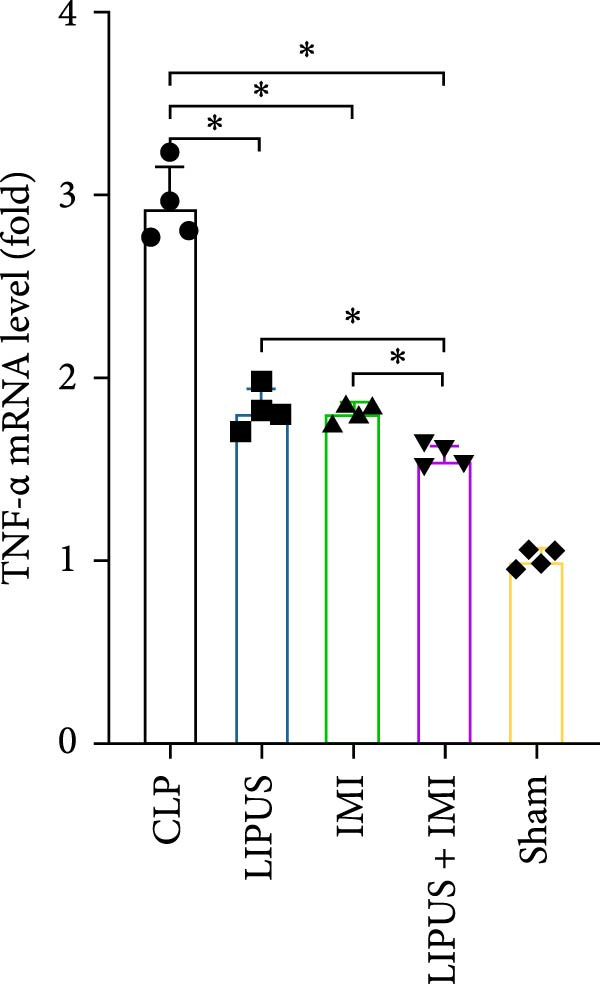
(A)

**Figure:**
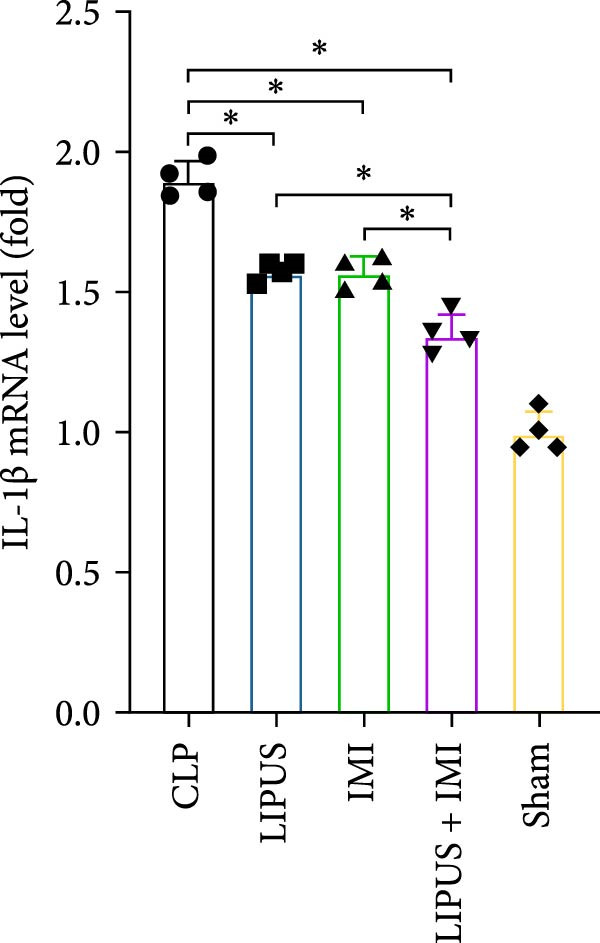
(B)

**Figure:**
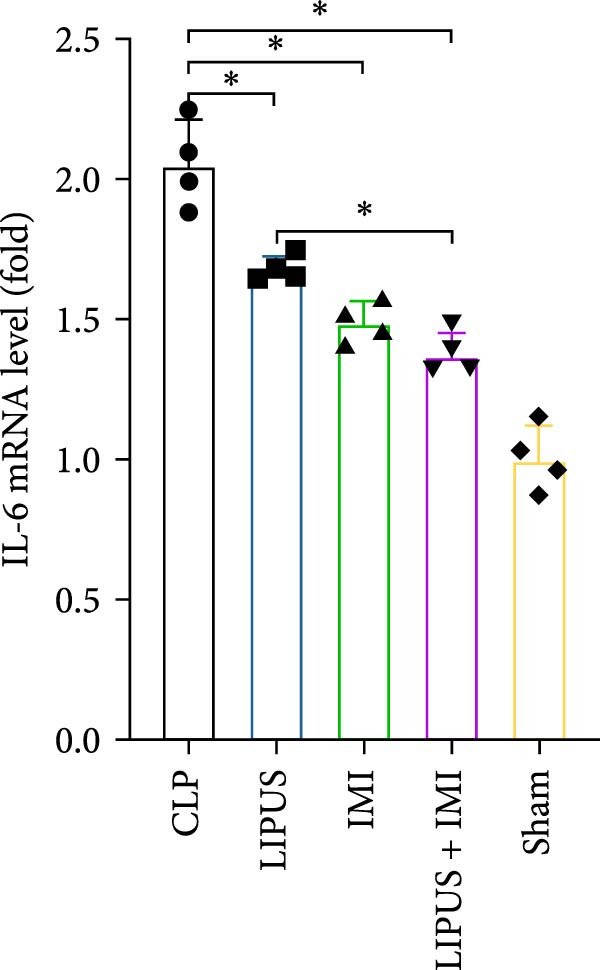
(C)

**Figure:**
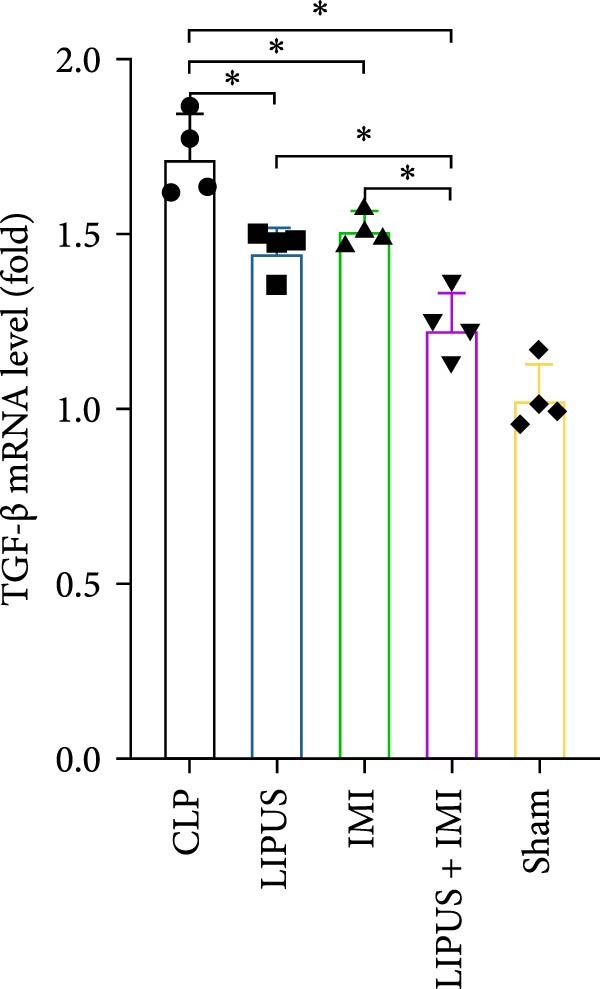
(D)

**Figure:**
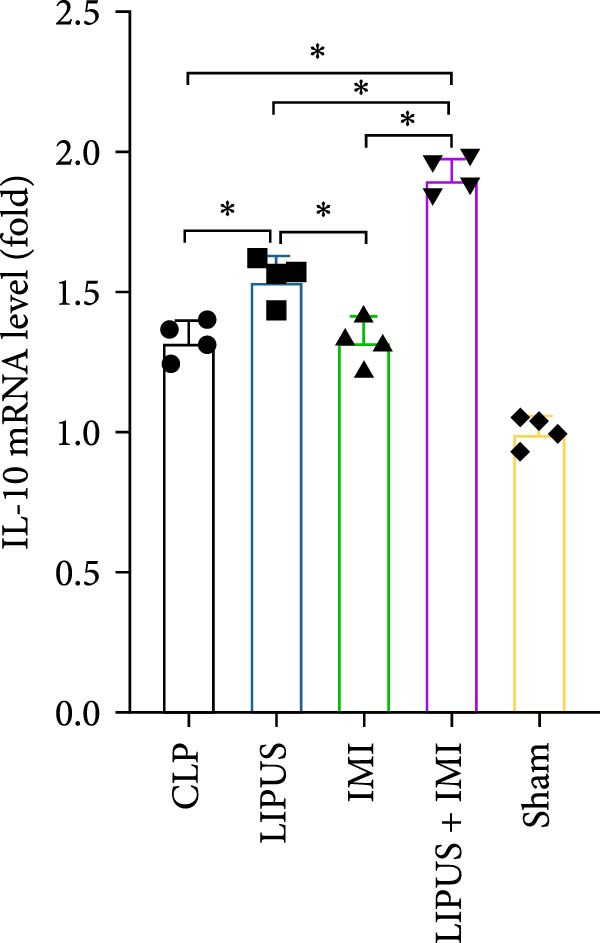
(E)

### 2.8. Effects of LIPUS Combined With IMI on Other Organs

As illustrated in Figure 6A, the Sham group exhibited normal structural and morphological characteristics in the heart, liver, lung, kidney, and ovary. In contrast, the CLP group displayed a range of pathological alterations: myocardial cell swelling, disorganized myofibril arrangement, and widened interstitial spaces in the heart; disorganized hepatocyte morphology, accumulation of red blood cells in hepatic sinusoids, and increased macrophage presence in the liver; interstitial edema, thickened alveolar walls, congested alveoli, and inflammatory cell infiltration in the lung; necrosis and sloughing of renal tubular epithelial cells along with renal interstitial congestion in the kidney; and interstitial edema and an increase in atretic follicles in the ovary. Notably, no significant damage was observed in these organs in the LIPUS, IMI, and LIPUS + IMI groups.

Figure 6Safety of LIPUS combined with IMI. (A) Effect of LIPUS combined with IMI on histopathology of heart, liver, lung, kidney and ovary. (B) Effect of LIPUS combined with IMI on the ultrastructure of heart, liver, lung, kidney, and ovary.

**Figure:**
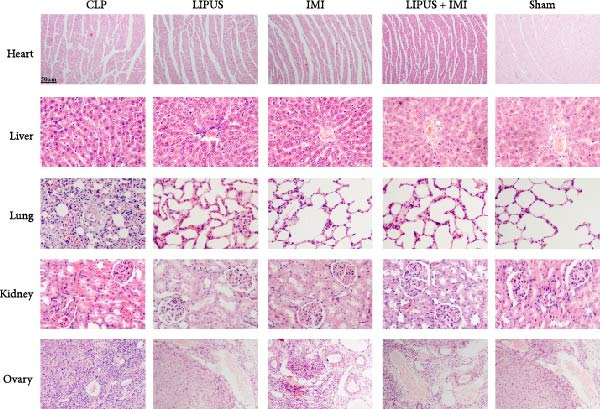
(A)

**Figure:**
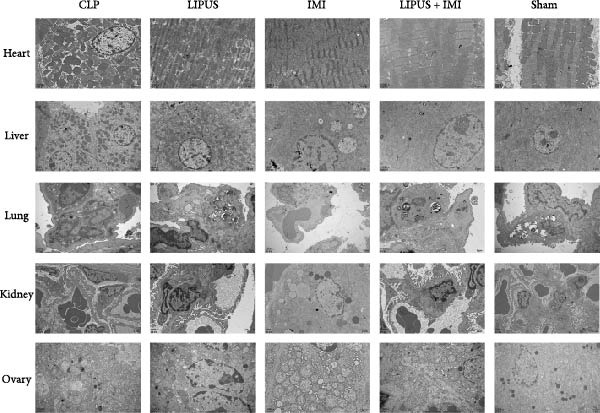
(B)

As illustrated in Figure 6B, the ultrastructural integrity of the heart, liver, lung, kidney, and ovary remained normal in the Sham group. In contrast, the CLP group exhibited a range of ultrastructural alterations: disorganized mitochondrial arrangement and myofilament disruption in cardiac tissue; mitochondria enveloped by rough endoplasmic reticulum, cytoplasmic edema, and dilated perinuclear space in hepatic cells; edema of type I alveolar cells and leukocyte occlusion within pulmonary capillary lumens; mitochondrial swelling and mesangial thickening in renal tissue; and mitochondrial swelling with evidence of autophagy in ovarian cells. Notably, no significant ultrastructural abnormalities were observed in the heart, liver, lung, kidney, and ovary in the LIPUS, IMI, and LIPUS + IMI groups, indicating that the combined treatment effectively mitigated sepsis‐induced organ damage.

## 3. Discussion

In this study, we evaluated the potential enhancement of therapeutic outcomes in septic rats through this combination therapy. The results indicated a significantly higher survival rate at 72 h for rats treated with both LIPUS and IMI compared to those treated with IMI alone. These findings suggest that the observed improvement may be related to the anti‐inflammatory effects of LIPUS, which could contribute to enhanced therapeutic outcomes.

Improving survival rates is a critical aspect of sepsis management [22]. In this study, the combination of LIPUS and IMI significantly enhanced survival within 72 h of sepsis in rats, highlighting the potential of this approach as a novel treatment option for sepsis. Currently, the early administration of antibiotics has become a widely accepted and biologically reasonable clinical strategy for improving survival outcomes in sepsis treatment [23, 24]. However, even a 1‐h delay in antibiotic treatment can result in absolute increases in mortality rates of 0.3%, 0.4%, and 1.8% for patients with sepsis, severe sepsis, and septic shock, respectively [25]. Despite their benefits, the excessive or inappropriate use of antibiotics can also lead to serious safety consequences, such as organ damage [26]. Therefore, it is crucial to optimize the timing of antibiotic administration in septic patients without compromising efficacy. IMI, a broad‐spectrum antibiotic capable of inhibiting most gram‐negative and positive bacteria as well as anaerobic bacteria, is considered an ideal choice [27]. The international campaign to save sepsis recommends early identification of sepsis patients and the immediate administration of antibiotics within 1 h of suspected sepsis and septic shock [28]. Consequently, in this study, IMI was initially administered 1 h after CLP surgery. However, the timing and frequency of subsequent doses are more crucial than the timing of the initial administration. Because IMI is chemically unstable and has a relatively short half‐life of approximately 0.4 days [29], it is typically administered every 6–8 h for 1–2 weeks in standard clinical regimens [10, 11]. Nevertheless, excessive use of antibiotics may lead to multiple organ dysfunction [30]. Previous research has suggested that combining LIPUS with IMI could exhibit synergistic anti‐inflammatory effects and may be a promising approach for early sepsis treatment [31]. PCT and CRP are widely utilized biomarkers in sepsis diagnosis [32, 33], and it was observed that their expression levels were lower in the LIPUS group compared to the CLP group from 4 to 72 h after treatment, suggesting that LIPUS may have anti‐inflammatory effects during this period.

Cytokine storm serves as another crucial determinant of survival in sepsis [34, 35]. Sepsis is initiated by the host immune response and excessive inflammation, leading to the release of pro‐inflammatory cytokines such as IL‐1*β*, IL‐6, and TNF‐*α* [36]. Excessive production of pro‐inflammatory cytokines can trigger a cytokine storm, which may result in multiple organ dysfunction and ultimately lead to death [37]. Previous studies have demonstrated that elevated levels of IL‐6 are significantly associated with CLP mortality [38], consistent with the findings observed in this study. We measured peak IL‐6 levels in rat spleen tissue 48 h after successful establishment of the CLP model. Therefore, we believe that this also accounts for the highest mortality rate within 48 h among all groups of rats. In contrast to early inflammatory mediators, HMGB1 plays a pivotal role as a late inflammatory mediator capable of activating intracellular NF‐*κ*B signaling pathway and exacerbating tissue damage; thus making it an important target for preventing and treating late‐stage sepsis [39, 40]. The results obtained from our current study support this notion. While IL‐1*β*, TNF‐*α*, and IL‐6 exhibited a declining trend following the initial peak within 72 h, implying potential resolution of the initial infection, the trajectory of HMGB1 levels remained inconspicuous and even continued to rise in the spleen until 72 h. However, combined treatment with LIPUS and IMI led to a significant reduction in HMGB1 levels, suggesting that this intervention could mitigate long‐term pathological consequences associated with sepsis induced by HMGB1. Henceforth, effective control of cytokine storm is imperative for sepsis patient survival. In this study, the combination therapy of LIPUS and IMI demonstrated potential anti‐inflammatory mechanisms during the 4–72 h timeframe post CLP operation, indicating its ability to attenuate cytokine storm and enhance survival rates. Further research is warranted to comprehensively elucidate underlying mechanisms and explore the full potential of this approach in managing cytokine storm among sepsis patients.

In a previous study [16], we discovered that LIPUS exhibited a protective effect against sepsis, potentially by inhibiting the IL‐1R/NF‐*κ*B signaling pathway and thereby mitigating inflammation. To further investigate whether the combination of LIPUS and IMI could also yield effective treatment through the IL‐1R/NF‐*κ*B signaling pathway in septic rats, additional experiments were conducted. The results indicated significant upregulation of TNF‐*α*, IL‐1R, IL‐1*β*, IL‐6, and NF‐*κ*B p65 expression levels in the CLP group; however, these protein levels significantly decreased following LIPUS + IMI intervention. Thus, it is plausible that LIPUS combined with IMI may exert a protective role during the early stages of sepsis by downregulating IL‐1*β* expression and inhibiting the NF‐*κ*B signaling pathway. However, it should be noted that these results are correlative, and further functional validation is needed to confirm the causal role of the IL‐1R/NF‐*κ*B signaling pathway in mediating the protective effects of LIPUS combined with IMI. Notably, an elevation in IL‐10 levels was observed in the LIPUS, IMI, and LIPUS + IMI groups. IL‐10 is generally regarded as an anti‐inflammatory cytokine that suppresses excessive immune activation and tissue injury during sepsis. IL‐10 elevation can help restore immune balance and limit cytokine storm–induced damage. Conversely, HMGB1 serves as a ubiquitous nuclear protein involved in transcriptional regulation under normal conditions [41]. Unlike early inflammatory mediators, such as TNF‐a, HMGB1 is considered to be a late mediator contributing to lethal systemic inflammation within cytokine‐mediated sepsis animal models [39]. Ulloa et al. [42] have demonstrated that ethyl pyruvate may potentially inhibit the release of early and late inflammatory mediators (TNF‐*α* and HMGB1) through modulation of the NF‐*κ*B signaling pathway. Our study revealed an initial elevation in serum and spleen levels of HMGB1 in septic rats induced by CLP, which were significantly attenuated by the combined intervention of LIPUS and IMI. Furthermore, inhibition of the TGF‐*β* signaling pathway has been shown to ameliorate organ dysfunction associated with sepsis and prolong survival in septic mice [43]. Previous research conducted by Pan et al. [44] found a significant reduction in hepatic expression levels of TGF‐*β* in septic rats treated with Dachengqi decoction, suggesting a potential anti‐inflammatory effect mediated through the TGF‐*β*/Smad3 signaling pathway. Consistent with these findings, our results showed a significant decrease in TGF‐*β* protein expression following combined LIPUS and IMI intervention, suggesting that this combination therapy significantly enhances survival rates and has the potential to reduce inflammation in septic rats.

## 4. Limitation

This study has several limitations. First, the antibiotic regimen requires further optimization, and we did not assess IMI pharmacokinetics or bacterial clearance, which limits mechanistic insights. Second, we lacked a “nonspleen” LIPUS control group (e.g., irradiation of a flank region), restricting interpretation of the regional specificity of ultrasound effects. Third, the hypothesis that splenic neuromodulation underlies the protective effects remains speculative without direct neuromodulatory evidence; future studies incorporating neural pathway blockade or electrophysiological verification are needed to clarify the mechanism. Fourth, the absence of splenectomy prior to CLP limits the ability to directly assess spleen involvement in sepsis progression. Fifth, LIPUS was applied only once; repeated or long‐term treatments may elicit different biological effects and warrant further investigation. Sixth, the 72‐h observation period may underestimate delayed mortality and later immunosuppressive phases of sepsis. Future studies will extend the observation period to 7 days and include surrogate markers of organ dysfunction such as ALT, creatinine, and lung histopathology scores to enhance translational value. Lastly, although rodent CLP models are widely used due to their advantages in size and sampling, they may not fully capture the heterogeneity and complexity of human sepsis, thus, caution is required when extrapolating findings to clinical settings. Future research should focus on large‐animal validation and clinical feasibility assessment of LIPUS therapy in septic conditions.

Although our current study provides preliminary evidence of the beneficial effects of combining LIPUS and IMI in mitigating inflammatory cytokine storms in septic rats, larger sample sizes and more in‐depth mechanistic investigations are needed to validate these findings. Future research may explore optimal parameters for LIPUS application and investigate the long‐term effects of this combination therapy on organ function and survival outcomes in sepsis.

## 5. Conclusion

In summary, the combination of LIPUS and IMI has been demonstrated to effectively treat septic rats potentially through inhibiting the IL‐1R/NF‐*κ*B signaling pathway and thereby reducing inflammation. The results of this study provide preliminary evidence that this combination therapy can optimize the strategy of antibiotic use in the treatment of sepsis, reduce organ damage, and ultimately improve survival outcomes. Overall, these findings suggest that LIPUS combined with IMI could represent a promising therapeutic approach to sepsis.

## 6. Materials and Methods

### 6.1. Experimental Animals

The study utilized 230 SPF female Sprague–Dawley (SD) rats, aged 6–8 weeks and weighing (200 ± 20) g, which were procured from the Laboratory Animal Center of Chongqing Medical University (License No.: SCXK [Chongqing] 2018‐0003). These rats were housed in a controlled SPF animal facility with a temperature of 22 ± 2°C, humidity of 45% ± 5%, normal day–night cycle, and ad libitum access to standard diet and water.

### 6.2. Sepsis Induction and Study Design

Following an adaptation period, rats were anesthetized with isoflurane (induction: ~4%–5%, maintenance: ~1.5%–2%, via inhalation), and the classic sepsis model was induced through CLP as described in previous studies [16, 45, 46]. A total of 80 SD rats were randomly allocated using a random number table by an investigator not involved in the experiments, and then assigned to five groups: immediate LIPUS treatment after CLP (LIPUS group; *n* = 16), IMI group (IMI group; *n* = 16), combined LIPUS and IMI treatment group (LIPUS + IMI group; *n* = 16), sepsis induced by CLP without intervention (CLP group; *n* = 16), and sham‐operated control group (Sham group; *n* = 16). All outcome assessments and data analyses were performed by researchers blinded to group allocation. The aforementioned five groups were utilized for the observation of survival within 72 h post‐modeling and treatment. The remaining 150 SD rats were randomly allocated into five groups for sampling at each designated time point: LIPUS group (*n* = 30, sampled at 4, 8, 16, 24, 48, and 72 h with a sample size of five each time), IMI group (*n* = 30, sampled at 4, 8, 16, 24, 48, and 72 h with a sample size of five each time), LIPUS + IMI group (*n* = 30, sampled at 4, 8, 16, 24, 48, and 72 h with a sample size of five each time), CLP group (*n* = 30, sampled at 4, 8, 16, 24, 48, and 72 h with a sample size of five each time), and Sham group (*n* = 30, sampled at 4, 8, 16, 24, 48, and 72 h with a sample size of five each time). The surgical procedures in the Sham group were consistent with those in the CLP group, except for the omission of CLP. All procedures conducted in this study were approved by the Experimental Animal Ethics Committee of Chongqing Medical University (No. 20180625).

### 6.3. Timing of IMI Implementation

To investigate the effects of LIPUS combined with IMI, we measured serum levels of PCT and CRP using enzyme linked immunosorbent assay (ELISA) kits (Jiangsu Jingmei Biotechnology Co., Ltd, China). This helped determine the optimal timing and frequency of IMI administration. The biomarkers PCT and CRP are widely acknowledged for their utility in sepsis diagnosis [4].

### 6.4. Treatment With LIPUS and IMI

LIPUS was provided by Chongqing Ronghai Engineering Research Center of Ultrasound Medical Co., Ltd, China. In the LIPUS and LIPUS + IMI groups, ultrasound irradiation was performed immediately after CLP surgery, targeting the spleen region. Ultrasound parameters were: 200 mW/cm^2^, 20 min, 0.37 MHz, 1 kHz repetition rate, and 20% duty cycle. The CLP and Sham groups received sham treatment. The IMI and LIPUS + IMI groups received IMI (25 mg/kg) at 1, 24, and 48 h post‐CLP, while other groups received normal saline (Qingzhou Yaowang Pharmaceutical Co., Ltd., China) at the same intervals.

### 6.5. Survival Analysis

In order to further compare the early survival of rats, 80 SD rats were randomly divided into LIPUS group, IMI group, LIPUS + IMI group, CLP group, and Sham group. After the establishment of sepsis model, the survival of SD rats was observed and recorded every 4 h after CLP operation. After 72 h of observation, the surviving SD rats were euthanized.

### 6.6. Sample Collection

The remaining 150 rats were used for collecting peripheral blood and tissue samples (heart, liver, spleen, lung, kidney, and ovary) at 4, 8, 16, 24, 48, and 72 h post‐CLP for further analysis.

### 6.7. Hematoxylin and Eosin (H&E) Staining of Tissue Samples

The heart, liver, spleen, lung, kidney, and ovary tissues of LIPUS group, IMI group, LIPUS + IMI group, CLP group, and Sham group were washed with normal saline and fixed in 5 mL centrifuge tube filled with 4% paraformaldehyde (Guangzhou Saiguo biotech Co., Ltd, Guangzhou, China) for 24 h. After dehydration, embedding, sectioning and HE staining, the pathological changes were observed under an optical microscope in five randomly selected fields (Olympus Co., Tokyo, Japan).

### 6.8. Transmission Electron Microscopy (TEM)

The heart, liver, spleen, lung, kidney, and ovary tissues of LIPUS group, IMI group, LIPUS + IMI group, CLP group, and Sham group were cut out 1 mm^3^ in size and put into 2.5% pentyl glycol fixative solution (Beijing Solaibao Technology Co. Ltd, Beijing, China). After embedding and staining, three fields were randomly selected under TEM Hitachi‐7500 to observe the ultrastructural changes (Hitachi, Tokyo, Japan).

### 6.9. ELISA

The spleens of rats collected at each time point from LIPUS group, IMI group, LIPUS + IMI group, CLP group, and Sham group were made into tissue homogenate. Both tissue homogenates and peripheral blood were centrifuged at 3000 rpm for 10 min at 4°C, and the supernatant was carefully collected and stored at −80°C. The levels of TNF‐*α*, IL‐1*β*, IL‐6, IL‐10, TGF‐*β*, and HMGB1 were detected using ELISA kits according to the manufacturer’s instructions (Jiangsu Jingmei Biotechnology Co., Ltd, Jiangsu, China).

### 6.10. Immunohistochemistry (IHC)

IHC was used to further study the effect of LIPUS combined with IMI on inflammatory factors in spleen tissue at each time point. Primary antibodies against IL‐1*β*, nuclear factor‐kappaB p65 (NF‐*κ*B p65), TNF‐*α*, IL‐6, and IL‐10 (Affinity Biosciences Ltd, OH, USA) were prepared according to the instructions of the immunohistochemical kit (Fuzhou Maixin Biotechnology Development Co., Ltd, Fuzhou, China). After adding appropriate primary antibody, the sections were incubated at 37°C for 1 h, then diaminobenzidine (DAB) was added, and five fields were randomly selected for observation under an optical microscope. Image J software (NIH, Bethesda, MD) was used to calculate the average optical density value of the positive area of each visual field for quantitative analysis.

### 6.11. Western Blot Analysis

Protein was extracted from the spleen tissue of rats in each group, and protein concentrations in each group were determined by bicinchoninic acid (BCA) protein assay kit (Beyotime, Beijing, China). Proteins were separated by 12% sodium dodecyl sulfate−polyacrylamide gel and transferred to polyvinylidene fluoride (PVDF) membranes (Merck KGaA, Darmstadt, Germany). The PVDF membranes were placed in 5% bovine serum albumin (BSA) solution and incubated for 1 h at 37°C on a shaker. After blocking, the appropriate primary antibodies against IL‐1*β*, IL‐1R, NF‐*κ*B p65, TNF‐*α*, IL‐6, IL‐10, TGF‐*β*, HMGB1, and *β*‐actin (Affinity Biosciences Ltd, OH, USA) were prepared according to the instructions, and the corresponding PVDF membranes were incubated with the primary antibodies at 4°C overnight. The next day, the PVDF membranes were incubated with HRP AffiniPure Goat Anti‐Rabbit IgG secondary antibody (Earthox, San Francisco, USA) for 1 h at 37°C on a shaker. Finally, the protein expression was imaged using a gel image analysis system (Syngene, Cambridge, UK) and quantitatively analyzed by ImageJ.

### 6.12. Quantitative Real‐Time Polymerase Chain Reaction (qRT‐PCR)

Total RNA was extracted from the spleen tissue of rats in each group using an RNA rapid extraction kit (Nanjing Vazyme Biology Co., Ltd, Nanjing, China), and the RNA concentration was detected after RNA elution. The extracted RNA of each group was used to synthesize cDNA according to the reverse transcription kit (TaKaRa, Tokyo, Japan), and then 20 μL of the reaction system was prepared for PCR amplification by referring to the fluorescence quantitative kit (Nanjing Vazyme Biology Co., Ltd, Nanjing, China). The qRT‐PCR reaction was performed according to the following conditions: preheating at 95°C for 5 min, followed by 5 s at 95°C, 30 s at 60°C, and 30 s at 72°C for a single cycle, and 40 cycles were repeated. TBP was used as an internal control, and the relative expression of IL‐1*β*, TNF‐*α*, IL‐6, IL‐10, and TGF‐*β* mRNA (Shanghai Shenggong Biological 9 Engineering Co., Ltd, Shanghai, China) was calculated by 2 ^−△△^ ct method. The primer sequences are shown in Table S1.

### 6.13. Statistical Analysis

All statistical analyses were performed using GraphPad Prism version 8 (GraphPad Software Inc., San Diego, CA). Survival differences within 72 h were analyzed using Kaplan–Meier survival curves followed by the log‐rank (Mantel–Cox) test. The remaining experimental data are presented as mean ± SD. ELISA and IHC data collected from different sets of animals at each time point were analyzed using two‐way factorial ANOVA (without repeated measures), followed by Tukey’s post hoc multiple comparisons test. WB and qRT‐PCR quantitative analyses were also performed using one‐way ANOVA with Tukey’s post hoc test. A *p*‐value <0.05 was considered statistically significant.

**Nomenclature**
BCA: Bicinchoninic acid
BSA: Bovine serum albumin
CLP: Cecal ligation and puncture
CRP: C‐reactive protein
ELISA: Enzyme linked immunosorbent assay
H&E: Hematoxylin and eosin
HMGB1: High mobility group protein box 1
IHC: Immunohistochemistry
IL‐6: Interleukin‐6
IL‐1R: Interleukin‐1 receptor
IL‐1*β*: Interleukin‐1 beta
IMI: Imipenem
LIPUS: Low‐intensity pulsed ultrasound
MODS: Multiple organ dysfunction syndrome
NF‐*κ*B: Nuclear factor kappa B
NF‐*κ*B p65: Nuclear factor‐kappa B p65
PCT: Procalcitonin
PVDF: Polyvinylidene fluoride
qRT‐PCR: Quantitative real‐time polymerase chain reaction
SD: Sprague–Dawley
TEM: Transmission electron microscope
TNF‐*α*: Tumor necrosis factor‐alpha
TGF‐*β*: Transforming growth factor‐beta.

## Ethics Statement

The process of animal experiment was carried out strictly in accordance with the standards of the Ethics Committee of the Animal Experiment Center of Chongqing Medical University (No. 20180625).

## Consent

The authors have nothing to report.

## Disclosure

All the authors have read and approved the final version of the manuscript.

## Conflicts of Interest

The authors declare no conflicts of interest.

## Author Contributions

Wentao Tang and Juan Deng designed and conducted the experiments, analyzed results, and drafted the manuscript with the assistance of Xinyi Zhang, Juan Qin, Guolin Song, and Chenghai Li. Xinyi Zhang was responsible for finalizing the manuscript and submitting it for publication. Xinfang Xiao, Liu Wu, and Yilin Tang performed the detection and measurement of experimental indicators. Xinyi Zhang, Yiqing Zhou, Junfen Li, and Sicheng He were responsible for data analysis and processing. Yan Wang conceived and supervised the study, and was responsible for the overall experimental design and data quality control. Wentao Tang and Juan Deng contributed equally to this work and shall share first authorship.

## Funding

This work was supported by the Program for Youth Innovation in Future Medicine, Chongqing Medical University (Grant W0155), the National Natural Science Foundation of China (Grant 12004059), and the Qiankehe Basic‐ZK [2024] General 415 Department of Science and Technology of Guizhou Province.

## Supporting Information

Additional supporting information can be found online in the Supporting Information section.

## Supporting information


**Supporting Information** Figure S1. Immunohistochemical analysis of the levels of cytokines (A–E) immunohistochemical staining (×400). Figure S2. Western blot (original image). Table S1. Primers used in the QRT‐PCR analysis.

## Data Availability

All the data generated or analyzed during this study are included in this published article.
