# Supplementary material for: Synergistic Alleviation of Inflammatory Cytokine Storms in Sepsis Rats by Low‐Intensity Pulsed Ultrasound and Imipenem
Source: Mediators Inflamm. 2026 Jan 16;2026:7323319. doi: 10.1155/mi/7323319 (PMC12809473; doi:10.1155/mi/7323319)
Supplement: Supplementary file 1 — Supporting Information Figure S1. Immunohistochemical analysis of the levels of cytokines (A–E) immunohistochemical staining (×400). Figure S2. Western blot (original image). Table S1. Primers used in the QRT‐PCR analysis. [file MI-2026-7323319-s001.zip › Table S1.docx]

**Table S1. PRIMERS USED IN THE QRT-PCR ANALYSIS**

| **Gene**  **(mouse)** | **Primer sequence** |
| --- | --- |
| IL-1β  TNF-α  IL-6  IL-10  TGF-β  TBP | F: GTGGCAGCTACCTGTGTCTT  R: CTCTGCTTGTGAGGTGCTGA  F: ATGGGCTCCCTCTCATCAGT  R: GGCTGGGTAGAGAACGGATG  F: GCCACTGCCTTCCCTACTTC  R: AGCACACTAGGTTTGCCGAG  F: GCTCAGCACTGCTATGTTGC  R: GTAGATGCCGGGTGGTTCAA  F: GACTCTCCACCTGCAAGACC  R: AGCCCTGTATTCCGTCTCCT  F: CTCAGTTACAGGTGGCAGCA  R: ACCAACAATCACCAACAGCA |

F: Forward primer; R: Revise primer
