# Supplementary figures and images for: Synergistic Alleviation of Inflammatory Cytokine Storms in Sepsis Rats by Low‐Intensity Pulsed Ultrasound and Imipenem
Source: Mediators Inflamm. 2026 Jan 16;2026:7323319. doi: 10.1155/mi/7323319 (PMC12809473; doi:10.1155/mi/7323319)

**FIGURE S2. WESTERN BLOT(ORIGINAL IMAGE)**


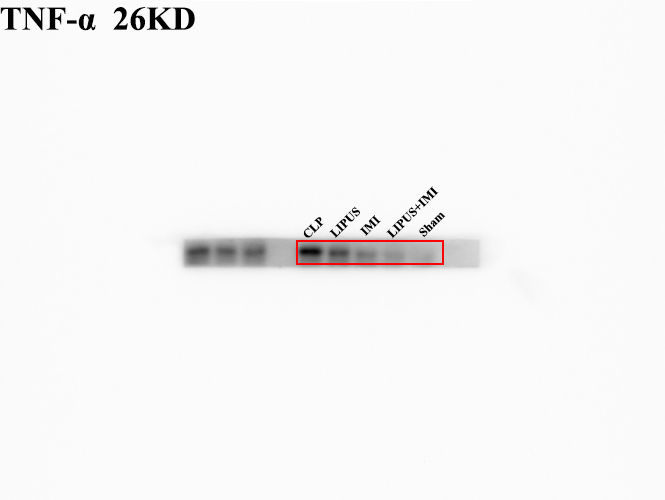


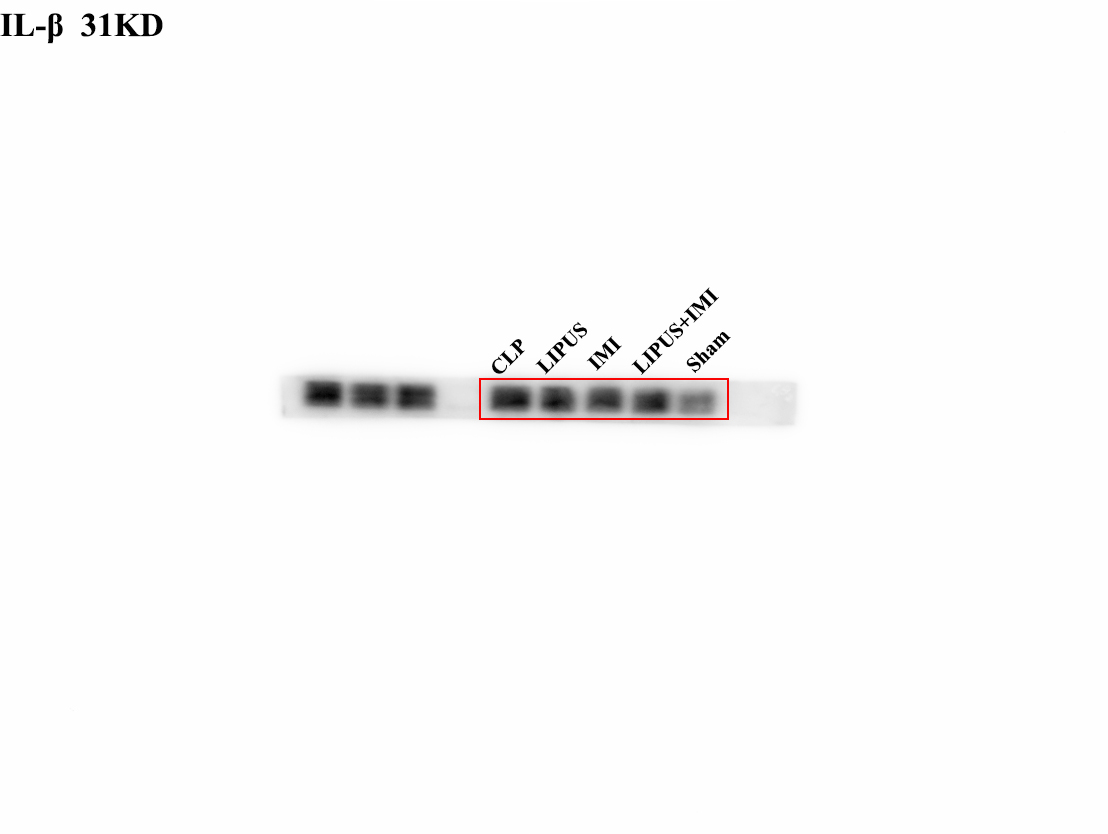


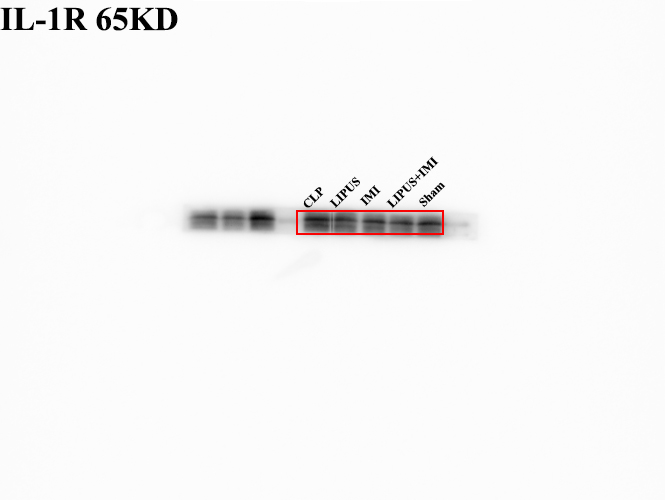


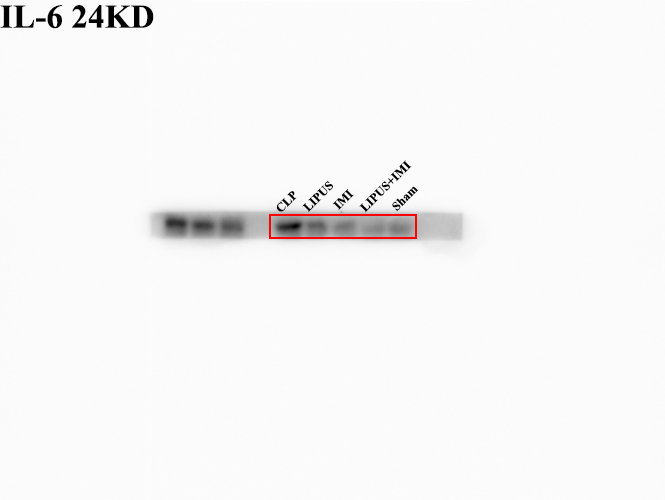


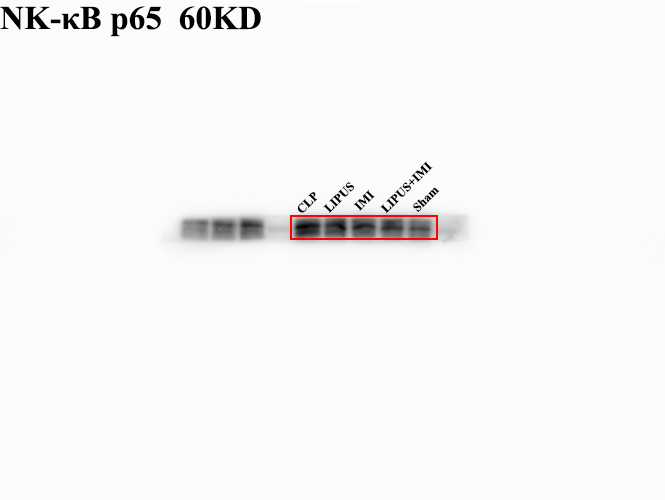


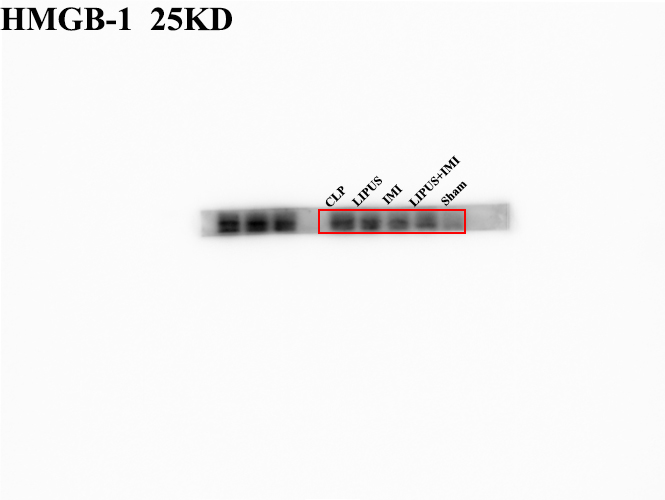


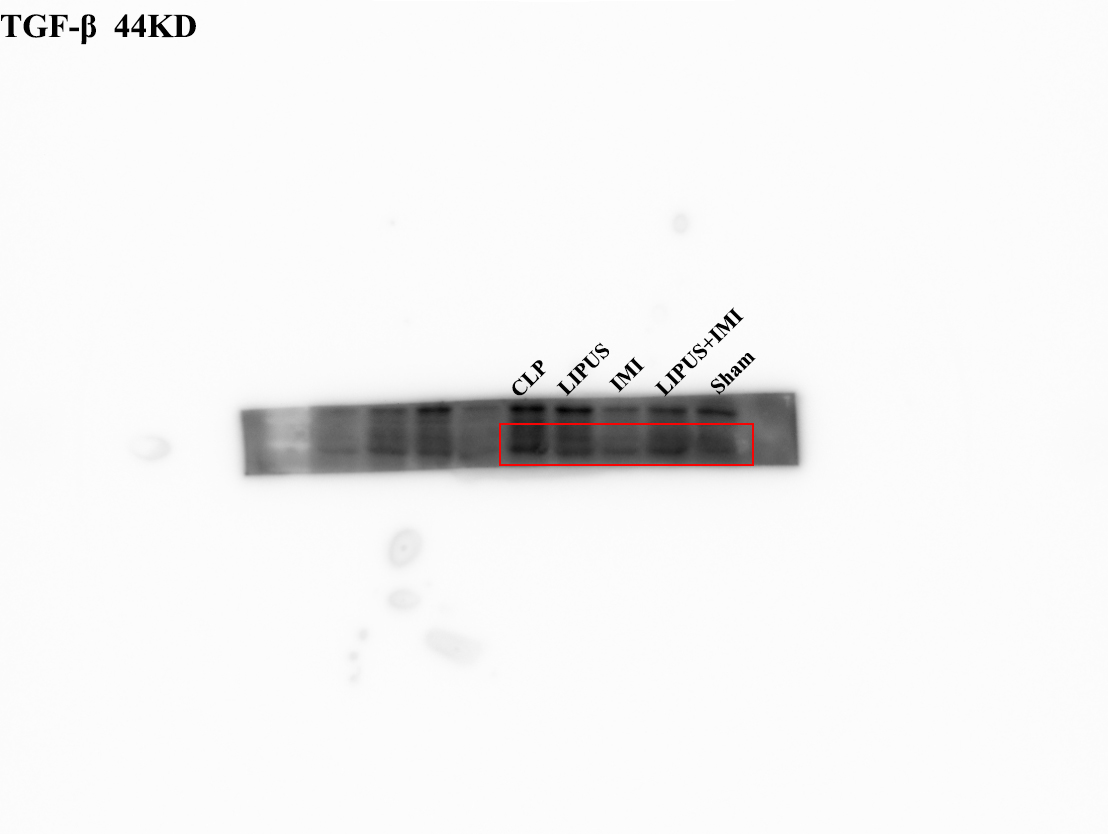


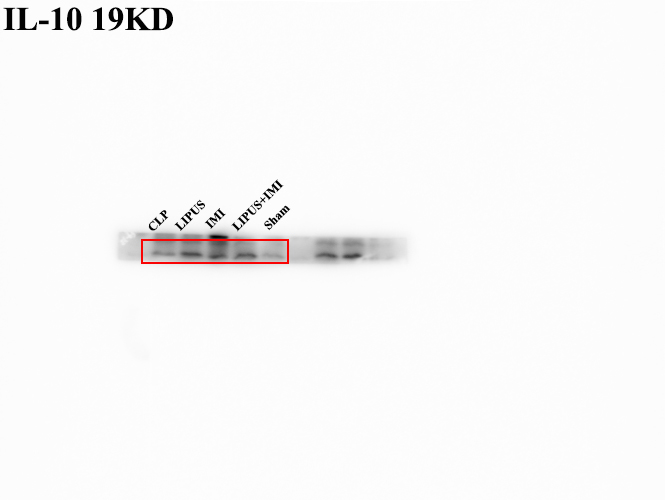


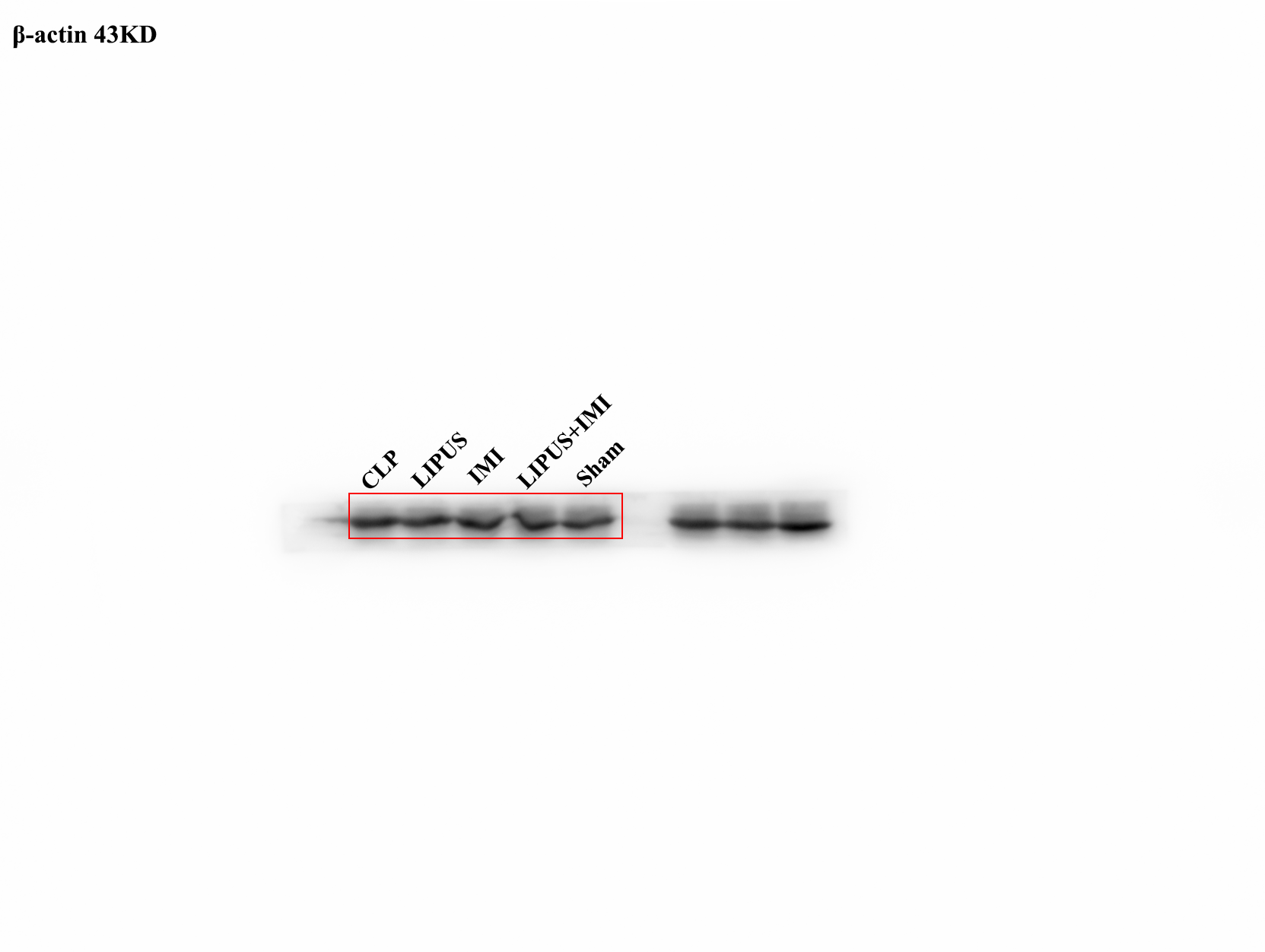

Supplement: Supplementary file 1 — Supporting Information Figure S1. Immunohistochemical analysis of the levels of cytokines (A–E) immunohistochemical staining (×400). Figure S2. Western blot (original image). Table S1. Primers used in the QRT‐PCR analysis. [file MI-2026-7323319-s001.zip › FIGURE S2(WB original image).docx]
